# Assessment of Natural Radioactivity Levels of Cements and Cement Composites in the Slovak Republic

**DOI:** 10.3390/ijerph10127165

**Published:** 2013-12-12

**Authors:** Adriana Eštoková, Lenka Palaščáková

**Affiliations:** Faculty of Civil Engineering, Institute of Environmental Engineering, Technical University of Košice, Vysokoškolská 4, Košice 042 00, Slovakia; E-Mail: lenka.palascakova@gmail.com

**Keywords:** cement, cement composites, natural radioactivity

## Abstract

The radionuclide activities of ^226^Ra, ^232^Th and ^40^K and radiological parameters (radium equivalent activity, gamma and alpha indices, the absorbed gamma dose rate and external and internal hazard indices) of cements and cement composites commonly used in the Slovak Republic have been studied in this paper. The cement samples of 8 types of cements from Slovak cement plants and five types of composites made from cement type CEM I were analyzed in the experiment. The radionuclide activities in the cements ranged from 8.58–19.1 Bq·kg^−1^, 9.78–26.3 Bq·kg^−1^ and 156.5–489.4 Bq·kg^−1^ for ^226^Ra, ^232^Th and ^40^K, respectively. The radiological parameters in cement samples were calculated as follows: mean radium equivalent activity *Ra_eq_* = 67.87 Bq·kg^−1^, gamma index *I_γ_* = 0.256, alpha index *I_α_* = 0.067, the absorbed gamma dose rate *D* = 60.76 nGy·h^−1^, external hazard index *H_ex_* = 0.182 and internal hazard index *H_in_* was 0.218. The radionuclide activity in composites ranged from 6.84–10.8 Bq·kg^−1^ for ^226^Ra, 13.1–20.5 Bq·kg^−1^ for ^232^Th and 250.4–494.4 Bq·kg^−1^ for ^40^K. The calculated radiological parameters of cements were lower than calculated radiological parameters of cement composites.

## 1. Introduction

The assessment of population exposures due to indoor radiation is very important and therefore knowledge of the concentrations of natural radionuclides in construction materials is required [1]. Construction materials are derived from both natural sources (e.g., rock and soil) and waste products (e.g., phosphogypsum, alum shale, coal fly ash, oil shale ash, some rare minerals, certain slags, *etc*.) and also from industry (e.g., power plants, phosphate fertilizer and oil industry) products. Although building materials act as a source of radiation to the inhabitants in their dwellings, they also have the role of a shield against outdoor radiation [2]. All building raw materials and products derived from rock and soil contain various amounts of mainly natural radionuclides of the uranium (^238^U) and thorium (^232^Th) series, and the radioactive isotope of potassium (^40^K). In the ^238^U series, the decay chain segment starting from radium (^226^Ra) is radiologically the most important and, therefore, reference is often made to ^226^Ra instead of ^238^U [3]. It has long been known that some construction materials are naturally more radioactive than others. The level of natural radioactivity in construction materials, even of low-level activity, gives rise to external and internal indoor exposure [4]. The external radiation exposure is caused by gamma radiation originating from members of the uranium and thorium decay chains and from ^40^K and the internal radiation exposure, mainly affecting the respiratory tract, is due to the short-lived daughter products of radon which are released from construction materials into room air [5]. Thus, the knowledge of radioactivity in building materials is important to estimate the radiological hazards to human health [6]. The most important naturally occurring radionuclides present in cements are ^226^Ra, ^232^Th and ^40^K, as mentioned above. Knowledge of basic radiological parameters such as radioactive contents and attenuation coefficients in building materials is important in the assessment of possible radiation exposure of the population as most people spend about 80% of their life inside houses and offices. This knowledge is essential for the development of standards and guidelines for the use of these materials [7].

The contents of ^226^Ra, ^232^Th and ^40^K in cements materials can vary considerably depending on their chemical composition in relation to geological source and geochemical characteristics [3]. Whereas in Greek Portland cements [8] the mass activity of ^226^Ra was measured at about 92 Bq·kg^−1^and in Malaysian cements at about 81.4 Bq·kg^−1^ [9]; Hizem *et al*. [10] mentioned lower activities of about 22 Bq·kg^−1,^ like [5] who measured ^226^Ra of 26.1 Bq·kg^−1^. A more complex approach to building materials radionuclide activity evaluation is based on the calculation of the corresponding radiological parameters.

In the study presented herein, the activities of natural radionuclides of ^226^Ra, ^232^Th and ^40^K, and radiological parameters such as radium equivalent activity, indoor absorbed dose rate, gamma and alpha indices and external and internal hazard indices were determined to assess the radiation risk associated with the cements and cement composites produced and commonly used in the Slovak Republic.

## 2. Materials and Methods

### 2.1. Sampling and Sample Preparation

The natural radioactivity was measured in eight types of cements produced in Slovak cement plants and in five types of composites made from the cement type of CEM I, which is also included in study. The number of samples analysed was at least four samples for each cement type and cement composite. The characterization of the assessed cement samples according to international standards [11] is presented in Table 1.

**Table 1:** The characteristics of assessed cement types.

Cement Type	Title	Composition
CEM I	Portland cement	Clinker in range of 95%–100%
CEM II/A-S	Portland slag cement	Portland cement and 6%–20% of slag
CEM II/B-S	Portland slag cement	Portland cement and 21%–35% of slag
CEM II/B-P	Portland puzzolanic cement	Portland cement and max 35% of natural puzzolana
CEM II/A-LL	Portland limestone cement	Portland cement and 6%–20% of limestone
CEM II/B-M	Portland composite cement	Portland cement and max 35% of slag, puzzolana, fly ash and limestone
CEM III	Blastfurnace cement	Portland cement and max 65% of slag
CEM V	Composite cement	Portland cement and more than 35% blastfurnace slag, puzzolana or fly ash

The cement composites (C1–C4) were prepared for the experiment in accordance with the standard procedure using CEM I cement [12]. The recipes of the prepared cement composites given for 1 m^3^ of composite are presented in Table 2.

**Table 2:** Mixture composition of tested composites.

Components		Samples Type			
		C1	C2	C3	C4
CEM I 42.5N (kg)		360	360	360	360
water (L)		170	198	197	205
zeolite (kg)		–	–	–	20
silica fume (kg)		–	–	20	20
aggregate (kg)	0/4 mm	825	825	800	750
	4/8 mm	235	235	235	235
	8/16 mm	740	740	740	740
plasticizer (L)		3.1	2.6	3.1	3.1
w/c		0.47	0.55	0.49	0.45

The recipe of C1 composite was similar to that of C2, but in the C2 recipe less plasticizer based on naphthalene-formaldehyde resins and more water were used, which resulted in a different water to cement ratio (w/c). The water to cement ratio was dealt with in order to meet the technical requirements and specifications of concrete. The samples prepared were cured during 28 days in a water environment. Then, several samples of set C2 were again immersed in the water (C2a) and others (C2b) were treated in a dry environment by covering with plastic wrap. After hardening the cement composites were crushed into powder form by using a MSK-SFM-1 Desk-Top planetary ball miller (MTI Corporation, Richmond, CA, USA) to study their chemical composition using X-ray fluorescence spectrometry. 

The cement and cement composite samples in powder form were prepared for X-ray fluorescence analysis (XRF) as pressed tablets (pellets) of 32 mm diameter by mixing of 5 g of sample and 1 g of dilution material and pressing with a hydraulic press applying a pressure of 10 tons during 60 s. 

The concentrations of natural radionuclides ^226^Ra, ^232^Th and ^40^K were measured in powdered cement samples (0.53 ± 0.07 kg) prepared in 450 mL size Marinelli containers. Thus, prepared samples in containers were sealed hermetically and stored for 40 days to achieve the secular equilibrium between ^226^Ra and its short-lived daughters before gamma spectrometry measurements. In cement composites, the concentrations of natural radionuclides ^226^Ra, ^232^Th and ^40^K were measured in compact samples. The secular equilibrium between ^226^Ra and its short-lived daughters before gamma spectrometry measurements was expected to be achieved in the composite hardening process.

### 2.2. Chemical Composition Measurements

The basic chemical composition of tested cements was investigated by X-ray fluorescence analysis using a SPECTRO iQ II (Ametek, Kleve, Germany) instrument equipped with a silicon drift detector (SDD) with resolution of 145 eV at 10,000 pulses. The primary beam was polarized by a Bragg crystal and Highly Ordered Pyrolytic Graphite–HOPG target. The samples were measured during 300 s at voltages of 25 and 50 kV at currents of 0.5 and 1.0 mA, respectively, under a helium atmosphere by using the standardized method of fundamental parameters for cement pellets.

### 2.3. Gamma Spectrometry Measurements

Measurement of radioactivity was carried out using an EMS-1A SH (Empos, Prague, Czech Republic) detection system equipped with a NaI/Tl scintillation detection probe and a MC4K multichannel analyzer with optimized resolution of 818 V, 4,096 channel and with 9 cm of lead shielding and internal lining of 2 mm tinned copper.

The spectra were first measured with empty containers (blank samples) and then with containers filled with weighed amounts of sample. The background of the detection system plays a vital role in the measurement of low-level activity as typically found in construction materials. The counting system must have a background as low as attainable with a minimum number of spectral lines originating from natural radionuclides which may be present in the system components and in the surrounding environment of the counting facility. In the presented study, routine measurements of the background count rates for natural radionuclides were carried out before each set of measurements, each for a counting time of 43,200 s (*i*.*e*., 12 h). The emphasis was on the determination of specific activity concentration of ^226^Ra, ^232^Th and ^40^K. The radioactivity of ^40^K was measured directly through its gamma ray energy peak at 1,460.8 keV, while activities of ^226^Ra and ^232^Th were calculated based on the mean value of their respective decay products. Activity of ^226^Ra was measured using the 351.9 keV gamma rays from ^214^Pb and the activity of ^232^Th was measured using the 238.6 keV gamma rays of ^212^Pb. Every sample was measured for 18,000 s.

## 3. Results and Discussion

### 3.1. Chemical Analysis

The percentage of basic components of tested cement samples and cement composites samples measured by XRF spectroscopy is shown in Table 3. The chemical composition of investigated cement samples correlate to the standard chemical composition of particular cement types [13]. The percentage of the oxides depends on both raw materials and constituents. 

The chemical composition of studied cement composites compared to the chemical composition of the tested Portland cements was similar except for their calcium and silicon contents. The content of SiO_2_ in cement was measured in the 17.8%–19.8% range and in cement composites it was increased from 25.97% to 45.63% due to the addition of aggregate, zeolite and silica fume (samples C3 and C4). The content of CaO in cement was from 54.2% to 63.6%, and in cement composites it was in the 25.12%–32.0% range. Differences in composition also depend on the structure of the composites as well as on the processes taking place in composite materials during hardening.

### 3.2. Activity Concentration

The measured specific activities of ^226^Ra, ^232^Th and ^40^K of the studied cements and cement composites are given in Table 4. The lowest mean value of ^226^Ra activity of the analyzed cement samples was measured in sample CEM II/A-LL Portland limestone cement (8.58 Bq·kg^−1^), while the highest mean value for the same radionuclide reaching 19.1 Bq·kg^−1^ was measured in CEM III-Blastfurnace cement (Table 4a). The highest activity mean value for ^232^Th (26.3 Bq·kg^−1^) was found in CEM V-Composite cement and the lowest one (9.78 Bq·kg^−1^) in CEM II/B-M-Portland composite cement. The ^40^K lowest mean value was 156.5 Bq·kg^−1^ measured in CEM I-Portland cement and the highest mean value was 489.4 Bq·kg^−1^ in CEM II/B-P-Portland puzzolanic cement. 

The contents of ^226^Ra, ^232^Th and ^40^K in tested cements depend on the raw materials and probably vary considerably in relation to the various geological source and geochemical characteristics. The average concentrations for radionuclides in Portland cements (CEM I) have been measured at 11.8 Bq·kg^−1^, 18.4 Bq·kg^−1^ and 156.5 Bq·kg^−1^ for ^226^Ra, ^232^Th and ^40^K, respectively. Radionuclide activities in CEM II cements reached slightly higher average values of 12.18 Bq·kg^−1^ for ^226^Ra, 18.6 Bq·kg^−1^ for ^232^Th and 354.3 Bq·kg^−1^ for ^40^K. Average activities of ^226^Ra and ^232^Th in CEM III cements have been observed to be even higher than in CEM I and CEM II cement samples (Table 4). 

The specific activity of ^226^Ra, ^232^Th and ^40^K determined in the presented study for Portland cements has also been compared with the values reported for Portland cements in other countries (Table 5). The measured activities due to all three radionuclides in Portland cements in Slovakia have been found to be comparable with those reported abroad.

Analysing the results of the specific activity due to ^226^Ra, ^232^Th and ^40^K for cement composites (Table 4b) the lowest mean values were measured in sample C4 (6.84 Bq·kg^−1^), in C2a (13.1 Bq·kg^−1^) and in C1 (250.4 Bq·kg^−1^) for ^226^Ra, ^232^Th and ^40^K, respectively. The highest mean values of ^226^Ra were measured in sample C2b (10.8 Bq·kg^−1^), of ^232^Th in C1 (20.5 Bq·kg^−1^) and of ^40^K in C2a (494.4 Bq·kg^−1^). The results showed that the specific activity due to ^40^K was the largest contributor to the total activity for all studied samples.

**Table 3:** The basic chemical composition of studied cements and cement composites.

Oxides (%)	CEM I	CEM II/A-S	CEM II/B-S	CEM II/B-P	CEM II/A-LL	CEM II/B-M	CEM III	CEM V	C1	C2a	C2b	C3	C4
MgO	1.54–3.82	4.23–4.72	5.62–5.83	2.06–2.37	2.04–2.05	2.19–2.35	4.88–8.45	3.75–4.94	3.04	2.87	2.96	2.38	2.73
Al_2_O_3_	3.87–4.39	4.31–4.80	5.46–5.57	6.91–8.04	4.68–4.68	4.51–5.08	5.13–7.20	7.18–9.57	5.21	4.53	5.03	5.25	5.39
SiO_2_	17.8–19.8	20.9–22.2	26.0–26.4	36.2–40.4	19.0–19.2	17.1–19.1	26.7–36.7	37.9–39.1	30.16	25.97	29.75	39.82	45.63
SO_3_	2.83–3.31	3.04–3.08	2.85–3.22	1.97–3.20	3.09–3.17	2.61–3.23	1.75–3.34	2.34–2.58	2.89	2.95	2.885	2.81	2.72
K_2_O	0.45–1.16	0.53–0.55	0.54–0.56	0.71–1.89	1.08–1.09	0.43–1.01	0.51–0.82	0.92–1.01	0.75	0.79	0.79	0.75	0.79
CaO	54.2–63.6	55.8–56.4	52.8–53.1	32.6–42.6	57.5–58.2	52.7–57.7	46.1–52.4	40.0–42.6	31.27	31.56	32.00	25.12	26.17
TiO_2_	0.21–0.26	0.21–0.22	0.26–0.30	0.26–0.51	0.21–0.22	0.21–0.47	0.23–0.37	0.24–0.32	0.27	0.26	0.26	0.27	0.26
MnO	0.03–0.38	0.35–0.36	0.38–0.45	0.09–0.62	0.04–0.33	0.07–0.33	0.24–0.49	0.35–0.38	0.38	0.38	0.37	0.38	0.36
Fe_2_O_3_	2.63–3.29	2.64–2.70	2.46–2.50	3.29–3.74	2.77–2.70	2.28–2.50	1.13–2.08	2.06–5.21	4.04	3.78	3.82	4.63	3.75

**Table 4:** Activity concentration of ^226^Ra, ^232^Th and ^40^K in studied cements and cement composites.

Sample	Activity Concentration (Bq·kg^−1^)					
	^226^Ra		^232^Th		^40^K	
	Range	Mean ± SD	Range	Mean ± SD	Range	Mean ± SD
*(a) Cement samples*						
CEM I	3.69–36.8	11.8 ± 9.0	11.8–24.9	18.4 ± 3.7	36.98–331.4	156.5 ± 101
CEM II/A-S	10.8–15.9	12.9 ± 2.6	18.9–32.8	23.9 ± 7.7	107.8–478.3	300.2 ± 186
CEM II/B-S	12.4–13.9	13.4 ± 0.8	17.1–34.2	23.8 ± 9.1	150.2–460.3	321.7 ± 158
CEM II/B-P	13.0–13.2	13.1 ± 0.1	16.7–26.7	21.7 ± 7.1	328.4–650.5	489.4 ± 228
CEM II/A-LL	8.19–8.98	8.58 ± 0.6	8.53–18.6	13.6 ± 7.2	178.9–516.0	347.5 ± 238
CEM II/B-M	12.1–13.8	12.9 ± 1.2	3.59–15.9	9.78 ± 8.8	145.9–479.9	312.9 ± 236
CEM III	15.8–23.3	19.1 ± 2.9	8.04–37.5	23.0 ± 9.5	111.3–452.3	293.3 ± 148
CEM V	14.6–20.9	18.7 ± 3.6	16.8–38.2	26.3 ± 11	219.6–733.7	397.2 ± 291
*(b) Cement composite samples*						
C1	6.11–12.6	9.38 ± 4.6	12.4–28.5	20.5 ± 11	227.3–273.5	250.4 ± 32.6
C2a	5.24–10.9	7.94 ± 2.8	3.96–24.9	13.1 ± 10	306.9–681.8	494.4 ± 265
C2b	8.03–13.5	10.8 ± 3.9	6.92–24.4	15.6 ± 12	310.3–340.8	325.6 ± 21.6
C3	6.33–8.98	7.65 ± 1.9	13.8–14.0	13.9 ± 0.2	301.8–473.7	387.7 ± 121
C4	5.88–7.88	6.84 ± 1.0	16.6–21.9	19.3 ± 2.5	261.0–366.8	313.9 ± 74.7

**Table 5:** Comparison of specific gamma activities (Bq·kg^−1^) of the Slovak Portland cement samples with that of other countries of the world.

Country	^226^Ra (Bq·kg^−1^)	^232^Th (Bq·kg^−1^)	^40^K (Bq·kg^−1^)	Reference
Australia	51.8	48.1	114.7	[14]
Austria	26.7	14.2	210	[15]
Bangladesh	61	80	1133	[16]
Brazil	61.7	58.5	564	[17]
China	51.7	32	207.7	[18]
Egypt	35	19	93	[19]
Finland	40.2	19.9	251	[20]
Greece	92	31	310	[8]
Italy	46	42	316	[21]
Japan	36	21	139	[22]
Malaysia	81.4	59.2	203.5	[9]
Netherlands	27	19	230	[23]
Norway	29.6	18.5	259	[24]
Pakistan	26.1	28.7	272.9	[5]
Turkey	41	26	267	[3]
Slovakia	11.8	18.4	156.5	Present study

**Figure 1:**
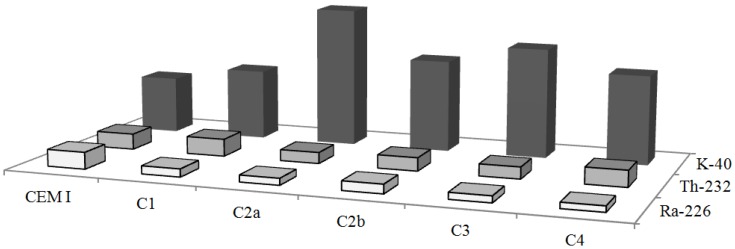
Comparison between the average values of ^226^Ra, ^232^Th and ^40^K activities for cement (used in composites) and cement composites.

The specific activities of ^226^Ra, ^232^Th and ^40^K of the cement composites were compared with specific activities of the cement which was used to produce them (Figure 1). The average concentrations of radionuclides in the Portland cement sample used to produce the composites, were 19.76 Bq·kg^−1^, 18.57 Bq·kg^−1^ and 203.36 Bq·kg^−1^ for ^226^Ra, ^232^Th and ^40^K, respectively. 

The ^226^Ra activities in all cement composites samples have been measured to be lower than in cement. On the contrary, the specific activities of ^40^K in cement composites have been measured to be higher than in cement for all investigated samples. The specific activities of ^232^Th reached higher values in C1 and C4 cement composites when compared to cement sample. 

Cement deserves further consideration because it is commonly used, together with inert aggregate materials, as a component of concrete or plaster, mortar and other surface materials. Since aggregate materials (typically sand and gravel from sedimentary rocks) have a low content of natural radionuclides, the major contribution to radioactivity in concretes is expected to come from the cement. For this reason one would expect activity concentrations to be systematically higher in cement than in concrete, but when we compared our data sets of these materials this hypothesis could not be definitely supported. There follows the need to recommend that the overall radioactivity of the concrete mixture should be controlled.

### 3.3. Radium Equivalent Activity (Ra_eq_)

The distribution of natural radionuclides in building materials samples under investigation is not uniform. Therefore, a common radiological index has been introduced to represent the specific radioactivity level of ^226^Ra, ^232^Th and ^40^K by a common index, which takes into account the radiation hazards associated with them. This index is usually known as radium equivalent (*Ra_eq_*) activity [14]:

*Ra_eq_ = A_Ra_+* 1.43*A_Th_* + 0.077*A_K_* (1)

where A_Ra_, A_Th_ and A_K_ are the specific activities of ^226^Ra, ^232^Th and ^40^K, respectively in Bq·kg^−1^. In the definition of Ra_eq_, it is assumed that 10 Bq·kg^−1^of ^226^Ra, 7 Bq·kg^−1^ of ^232^Th and 130 Bq·kg^−1^ of ^40^K produce equal gamma-ray dose rate [14].

The range of radium equivalent activities *Ra_eq_* was calculated for all assessed cement samples from 51.05–86.87 Bq·kg^−1^ (Table 6a). From the results it can be noticed that the lowest value of *Ra_eq_* (51.05 Bq·kg^−1^) was calculated for CEM II/B-M-Portland composite cement, while the highest value of 86.87 Bq·kg^−1^ was calculated for CEM V-Composite cement.

**Table 6:** The average values of radiation hazard parameters for studied cement and cement composites.

Sample	*Ra_eq_* (Bq·kg^−1^)	*D* (nGy·h^−1^)	*Iγ*	*I_α_*	*H_ex_*	*H_in_*
*(a) Cement samples*						
CEM I	51.33 ± 14.9	44.55 ± 14.6	0.188 ± 0.06	0.060 ± 0.04	0.139 ± 0.04	0.171 ± 0.07
CEM II/A-S	70.36 ± 16.7	62.31 ± 15.8	0.263 ± 0.07	0.065 ± 0.01	0.190 ± 0.05	0.225 ± 0.04
CEM II/B-S	72.18 ± 22.1	64.22 ± 19.8	0.271 ± 0.09	0.067 ± 0.01	0.195 ± 0.06	0.231 ± 0.06
CEM II/B-P	81.87 ± 7.47	75.11 ± 10.4	0.315 ± 0.04	0.066 ± 0.01	0.221 ± 0.02	0.256 ± 0.02
CEM II/A-LL	54.77 ± 8.68	50.64 ± 11.7	0.212 ± 0.05	0.043 ± 0.01	0.148 ± 0.02	0.171 ± 0.03
CEM II/B-M	51.05 ± 6.85	45.05 ± 15.4	0.196 ± 0.04	0.043 ± 0.02	0.130 ± 0.05	0.153 ± 0.05
CEM III	74.54 ± 15.6	66.31 ± 14.0	0.276 ± 0.06	0.095 ± 0.02	0.201 ± 0.04	0.253 ± 0.04
CEM V	86.87 ± 15.9	77.89 ± 19.6	0.326 ± 0.13	0.094 ± 0.02	0.235 ± 0.09	0.285 ± 0.08
*(b) Cement composites samples*						
C1	57.90 ± 9.18	51.15 ± 5.69	0.217 ± 0.03	0.047 ± 0.02	0.156 ± 0.03	0.182 ± 0.01
C2a	66.75 ± 3.24	62.83 ± 8.60	0.264 ± 0.03	0.040 ± 0.02	0.180 ± 0.01	0.202 ± 0.02
C2b	58.22 ± 12.1	53.17 ± 8.26	0.223 ± 0.04	0.054 ± 0.02	0.157 ± 0.03	0.186 ± 0.02
C3	57.37 ± 7.26	53.34 ± 7.83	0.224 ± 0.03	0.038 ± 0.01	0.155 ± 0.02	0.176 ± 0.02
C4	55.65 ± 3.14	50.36 ± 3.75	0.214 ± 0.02	0.034 ± 0.02	0.150 ± 0.01	0.169 ± 0.01

The high *Ra_eq_* values calculated for CEM II/B-P-Portland puzzolanic cement and CEM V/A-Composite cement can be due to the high concentration of the three radionuclides ^226^Ra, ^232^Th and ^40^K in these materials, as shown in Table 4. Comparing the results, it is evident that there are considerable variations in the *Ra_eq_* of different cement types. This fact is important from the point of view of selecting suitable cements for use in buildings and construction, especially concerning those which have large variations in their activities. Large variation in radium equivalent activities may suggest that it is advisable to monitor the radioactivity levels of cements from a new source before adopting it for use as a building material. The maximum value of *Ra_eq_* in building raw materials and products must be less than 370 Bq·kg^−1^ for safe use, *i*.*e*., to keep the external dose below 1.5 mSv·y^−1^ [25]. Radium equivalent activities of all assessed cement samples have been calculated to be lower than 370 Bq·kg^−1^, what means using these cements as building materials is safe. 

**Figure 2:**
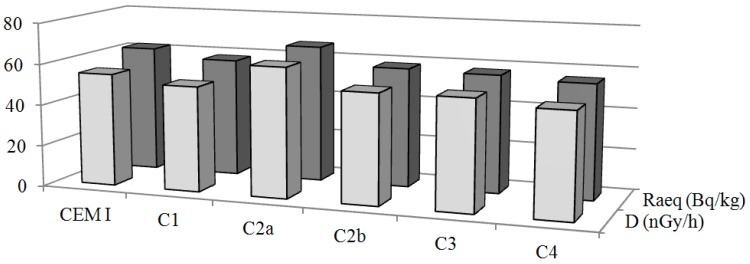
Comparison between the average values of radium equivalent *Ra_eq_* and absorbent dose rate *D* for cement (used in composites) and cement composites.

The mean *Ra_eq_* values calculated for cement composites are shown in Table 6b. The minimum (55.65 Bq·kg^−1^) and the maximum (66.75 Bq·kg^−1^) values of *Ra_eq_* were found in sample C4 and C2a of cement composites, respectively. The mean values of all measured cement composites samples were almost six times lower than the limit value of 370 Bq·kg^−1^ [25]. Comparing *Ra_eq_* of cement composites to *Ra_eq_* of cement used to their production, the higher value was found out only in sample C2 (Figure 2).

### 3.4. Estimation of the Absorbed Gamma Dose Rate

There is concern that some of the buildings will cause excessive radiation doses to the total body due to gamma rays emitted by ^214^Pb and ^214^Bi progeny of ^226^Ra and ^232^Th decay chains, and ^40^K also contributes to the total body radiation dose. The absorbed dose rate in indoor air due to gamma-ray emission from activity concentrations of ^226^Ra, ^232^Th and ^40^K was estimated using Equation (2) provided by [25,26], in which the dose conversion coefficients were calculated for the standard room centre. Dimensions of the room were 4 m × 5 m × 2.8 m. Thicknesses of walls, floor and ceiling and density of the structures were 20 cm and 2,350 kg·m^−3^ (concrete), respectively. The conversion factor used for calculation of the absorbed gamma dose rate D (nGy·h^−1^) corresponds to 0.92 nGy·h^−1^ per Bq·kg^−1^ for ^226^Ra, 1.1 nGy·h^−1^ per 1 Bq·kg^−1^ for ^232^Th and 0.08 nGy·h^−1^ per 1 Bq·kg^−1^ for ^40^K [26]:

*D* = 0.92*A_Ra_* + 1.1*A_Th_* + 0.08*A_k _* (2)

where A_Ra_, A_Th_ and A_K_ are the specific activities of ^226^Ra, ^232^Th and ^40^K, respectively in Bq·kg^−1^. The estimated indoor gamma dose rate values for cement and cement composites are also shown in the second column of Table 6. The *D* mean values for cement range from 44.55 to 77.89 nGy·h^−1^ and for cement composites range from 50.36 to 62.83 nGy·h^−1^. The mean values of *D* from all studied samples are lower than the world average (population-weighted) indoor absorbed gamma dose rate of 84 nGy·h^−1^ [26]. Figure 2 shows the comparison of absorbent dose rate between cement and cement composites. The values of *D* in cement composites are less than value of *D* in cement from which they made, except of value of sample C2a. This fact is caused probably due to high value of specific activity ^40^K in sample C2a.

### 3.5. Gamma Index

In order to assess whether the safety requirements for building materials are being fulfilled, a gamma index *I_γ_ i*s calculated as proposed by the European Commission [26]:

*I_γ_* = *A_Ra_/300* + *A_Th_/200* + *A_k_/3000* (3)

where A_Ra_, A_Th_ and A_K_ are the specific activities of ^226^Ra, ^232^Th, and ^40^K, respectively in Bq·kg^−1^. *I_γ_* ≤ 1 corresponds to an absorbed gamma dose rate less or equal to 1 mSv·y^−1^, while *I_γ_* ≤ 0.5 corresponds to a dose rate less or equal to 0.3 mSv·y^−1^ [26].

The mean values of index of natural radionuclide mass activity (gamma index, *I_γ_*) calculated from the measured activity concentration of ^226^Ra, ^232^Th and ^40^K are presented in the third column of Table 6 for cements and cement composites. The mean calculated values of *I_γ_* for the studied samples values varied in the range between 0.188–0.326 and 0.214–0.264 for cement and cement composites, respectively. 

The lowest value of *I_γ_* was calculated at 0.188 for CEM I-Portland cement and at 0.214 for cement composites in sample C4 and the highest ones were calculated at 0.326 for CEM V-Composite cement and 0.264 for C2a. The gamma index should also take into account typical ways and amounts in which the material is used in a building. The limit values depend on the dose criteria, the way and amount of the material and the manner in which it was used in a building and construction. For material used in bulk amounts *I_γ_* ≤ 1 corresponds to an absorbed gamma dose rate of 1 mSv·y^−1^ [26]. The gamma index calculated for all assessed samples was less than gamma index limit.

**Figure 3:**
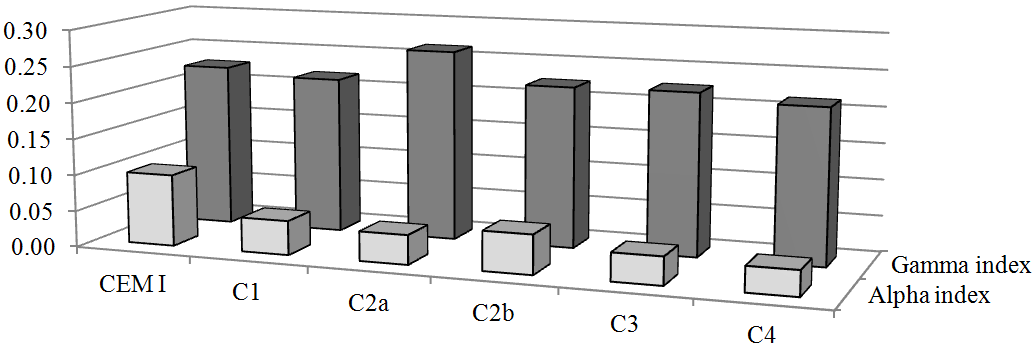
Comparison between the average values of gamma and alpha index of cement (used in composites) and cement composites.

The mean values of gamma index calculated for the cement composites were compared with mean values of gamma index calculated for cement sample, which was used to produce them (Figure 3). The values of *I_γ_* calculated for cement composites are less than value of *I_γ_* calculated for cement from which they have been made, except for value of sample C2a.

### 3.6. Alpha Index

Assessment of the internal hazard, originating from the alpha activity of building materials, requires calculations of the alpha index or internal hazard index. The alpha indices have been proposed to assess the exposure level due to radon inhalation originating from building materials [26]. The alpha index was determined by the following formula:

*I_α_* = *A_Ra_/*200 (4)

where *A_Ra_* is the activity concentration of ^226^Ra in Bq·kg^−1^ assumed in equilibrium with ^238^U. The safe use of materials in building construction requires *I_α_* to be less than 1. This limit corresponds to the ^226^Ra concentration of 200 Bq·kg^−1^ for building construction. The recommended exemption and recommended upper levels of ^226^Ra concentrations in building materials are 100 Bq·kg^−1^ and 200 Bq·kg^−1^. When the ^226^Ra activity concentration of building materials exceeds the value of 200 Bq·kg^−1^, it is possible that radon exhalation from this material may cause indoor radon concentration greater than 200 Bq·m^−3^. On the other hand, if ^226^Ra concentration is less than 100 Bq·kg^−1^, than resulting indoor radon concentration would be less than 200 Bq·m^−3^ [27]. 

The mean values of *I_α_* for the different cement samples in this study are summarised in the fourth column of Table 6. The maximum calculated values of *I_α_* for cements (CEM III-Blastfurnace cement/CEM III-Blastfurnace cement) of 0.095 and cement composites (sample C2b) of 0.054 were below the limits for safe use. When compared the *I_α_* of cement composites with *I_α_* of cement used to their production all *I_α_* values of cement composites have been calculated to be lower than in cement (Figure 3).

### 3.7. External Hazard Index

In order to assess the external radiological hazards from building materials, external hazard index (*H_ex_*) is calculated using the following formula [14]:

*H_ex_* = *A_Ra_/370* + *A_Th_/259* + *A_K_/4810* (5)

where A_Ra_, A_Th_ and A_K_ are the specific activities of ^226^Ra, ^232^Th, and ^40^K, respectively in Bq·kg^−1^. The value of this index must be less than unity for the radiation hazard to be negligible, *i*.*e*., the radiation exposure due to radioactivity in construction materials must be limited to 1.5 mSv·y^−1^. Then *H_ex_* should obey the following relation *H_ex_* ≤ 1 [28]. The mean values of *H_ex_* for the different samples in this study are shown in the fifth column of Table 6. The mean values of external hazard index for the cement and cement composites samples varied from 0.130 (CEM II/B-M-Portland composite cement) to 0.235 (CEM V-Composite cement) and from 0.150 (C4 cement composite) to 0.180 (C2a cement composite). The external hazard index for the studied samples is less than unity and therefore these building materials are safe to be used for construction.

### 3.8. Internal Hazard Index

In addition to the external irradiation, radon and its short-lived products are also hazardous to the respiratory organs. The internal exposure to radon and its daughter products is quantified by the internal hazard index (*H_in_*) which is given by the following formula [14]:

*H_in_* = *A_Ra_/185* + *A_Th_/259* + *A_K_/4810* (6)

where A_Ra_, A_Th_ and A_K_ are the specific activities of ^226^Ra, ^232^Th, and ^40^K, respectively in Bq·kg^−1^. For the safe use of a material in the construction of dwellings *H_in_* should be less than unity [28]. 

The lowest mean values of external hazard indices for the cement and cement composites samples were calculated in samples CEM II/B-M-Portland composite cement (0.153) and in C4 (0.169) and highest mean values were calculated in samples CEM V-Composite cement (0.285) and in C2a (0.202) (Table 6). The internal hazard index for the studied samples of building materials has been calculated to be lower than unity which indicates that the studied building materials are safe to be used for construction.

**Figure 4:**
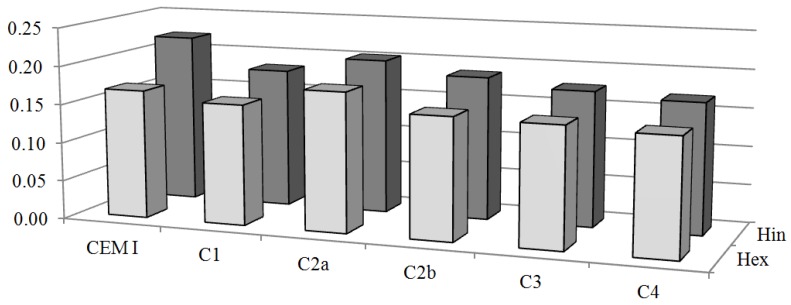
Comparison between the average values of internal and external hazard index of cement (used in composites) and cement composites.

The values of *H_in_* and *H_ex_* calculated for cement composites and cement from which are they made are shown in Figure 4. It can be seen that the values of *H_in_* and *H_ex_* in cement composites are less than values of *H_in_* and *H_ex_* in cement from which they made, except for the value of sample C2a. This fact is likely due to high value of ^40^K specific activity in cement composite sample C2a. The values of *H_in_* and *H_ex_* calculated for all studied samples are below the limits for safe use.

## 4. Conclusions

The natural radionuclide content of the ^226^Ra, ^232^Th, and ^40^K in the cement samples produced by Slovak producers and in cement composites were measured by using the technique of gamma-ray spectroscopy. The results may be useful in the assessment of the exposures and the radiation doses due to the natural radioactive element contents in cement samples.

- The mean activity concentrations of ^226^Ra, ^232^Th, and ^40^K were 13.82, 20.07 and 327.36 Bq·kg^−1^ in cement samples and 8.52, 16.47 and 354.39 Bq·kg^−1^ in cement composites samples, respectively. A comparison of the concentrations obtained in the study in Slovakia with the results abroad indicates that the radioactivity content of the Portland cement samples was quite lower, but it is not significantly different.
- The calculated mean radium equivalent activity (*Ra_eq_* = 67.87 Bq·kg^−1^), gamma index (*Iγ* = 0.256), alpha index (*I_α_* = 0.067), the absorbed gamma dose rate (*D* = 60.76 nGy·h^−1^), external hazard index (*H_ex_* = 0.182) and internal hazard index (*H_in_* = 0.218) in cement samples were lower than the recommended limits.
- The calculate radiological parameters *Ra_eq_, Iγ, I_α_, D, H_ex_* and *H_in_* in composites samples were 59.18 Bq·kg^−1^, 0.228, 0.043, 54.17 nGy·h^−1^, 0.160 and 0.183, respectively. These values were lower than the recommended limits, therefore, the use of these concrete materials in the construction of dwellings is considered to be safe for the dwellers.
- The calculated radiological parameters of cement composites were lower than values calculated for cement from which they made, except for values of *Ra_eq_*, *Iγ*, D and *H_ex_* calculated in sample C2a, which were higher than in cement. This is probably due to high value of ^40^K specific activity in sample C2a.

The results of the present study could be a valuable database for future estimations of the impact of radioactive pollution as well as for the improving of the specific requirements for cements in the Slovak eco-labelling process.
